# Did aculeate silk evolve as an antifouling material?

**DOI:** 10.1371/journal.pone.0203948

**Published:** 2018-09-21

**Authors:** Tara D. Sutherland, Alagacone Sriskantha, Trevor D. Rapson, Benjamin D. Kaehler, Gavin A. Huttley

**Affiliations:** 1 CSIRO (The Commonwealth Scientific and Industrial Research Organisation), Health and Biosecurity, Canberra, Australian Capital Territory, Australia; 2 Research School of Biology, Australian National University, Australian Capital Territory, Australia; Max-Planck-Institut fur Kolloid und Grenzflachenforschung, GERMANY

## Abstract

Many of the challenges we currently face as an advanced society have been solved in unique ways by biological systems. One such challenge is developing strategies to avoid microbial infection. Social aculeates (wasps, bees and ants) mitigate the risk of infection to their colonies using a wide range of adaptations and mechanisms. These adaptations and mechanisms are reliant on intricate social structures and are energetically costly for the colony. It seems likely that these species must have had alternative and simpler mechanisms in place to ensure the maintenance of hygienic domicile conditions prior to the evolution of these complex behaviours. Features of the aculeate coiled-coil silk proteins are reminiscent of those of naturally occurring α-helical antimicrobial peptides (AMPs). In this study, we demonstrate that peptides derived from the aculeate silk proteins have antimicrobial activity. We reconstruct the predicted ancestral silk sequences of an aculeate ancestor that pre-dates the evolution of sociality and demonstrate that these ancestral sequences also contained peptides with antimicrobial properties. It is possible that the silks evolved as an antifouling material and facilitated the evolution of sociality. These materials serve as model materials for consideration in future biomaterial development.

## Introduction

Although sociality provides many advantages, living in a social environment intensifies the risk of infection in comparison to non-social living. In the natural world, there are many examples of social living, with some of the most densely populated social communities occurring in insects. Social insects living at high density face an increased probability of acquiring infection through contact with other individuals [1]. Insect colonies contain many genetically-related individuals, and therefore disease susceptibility of an individual will be reflected throughout the colony [1]. In addition, social behaviour such as the exchange of food between individuals promotes infection transmission [1]. Furthermore, colonies require sufficient resources to sustain large populations and the environments that provide these will also be inhabited by a diverse community of microbes, including pathogens. As such, animals in these colonies are more likely to come into contact with pathogens than their less social counterparts [1].

Sociality has arisen independently many times within the aculeates [2,3], a monophyletic subclade of Hymenopteran insects that are characterized by the ability to deliver a venomous sting. Each of these social groups has evolved a wide range of behavioural and physiological adaptations and spatial mechanisms to mitigate the risk of infection associated with social living [4]. The adaptations and mechanisms that are manifest in the colonies of these species are generally reliant on the intricate social structure present in the colony [5]. Hence, prior to the evolution of this social structure, the ancestors of these species must have had alternative simpler mechanisms in place to ensure maintenance of hygienic domicile conditions in order to allow the evolution of these complex social structures.

The cocoons, hives or nests of social aculeate species are constructed using a distinctive silk, characterised by a coiled-coil molecular structure [6,7]. A completely different set of silk proteins is used in producing the cocoons of the Chrysidoidea (parasitic wasps), also within the aculeate subclade. These proteins predominantly adopt a β-sheet structure similar to that of the silk proteins that evolved convergently in spiders and silkworms [6,8]. The Chrysidoidea silk is considered basal to the Hymenoptera [8]. Interestingly, sociality has not evolved in any species within the Chrysidoidea. The co-incidental evolution of sociality (on multiple occasions) only within lineages that have evolved the ability to produce the coiled-coil structured silk raises the possibility of a link between use of this particular silk structure and evolution of sociality in aculeates.

The coiled-coil silk produced by the Vespoidea (hornets and wasps), Apoidea (sphecoid wasps and bees) and Formicoidea (ants) is encoded within four homologous genes. Phylogenetic analysis suggests that a single gene was duplicated three times in the ancestor of these lineages and a single copy of each paralogue has been retained in all extant species [6,9–11]. Both the basal β-sheet structured silk and the coiled-coil structured silk are produced from modified labial glands [8]. The simplest explanation for the evolution of the coiled-coil silk from within the background of the basal silk is that there was a loss of the gene(s) encoding the β-sheet structured silk and gain in the gene encoding the coiled-coil silk. Such a rare event suggests the functional properties of the new gene encoding the coiled-coil protein conferred an enormous selective advantage to the early aculeates.

The naturally occurring coiled-coil silk is tougher and retains its properties when wet in comparison to other silks [12]. For social species that need to construct long-lasting domiciles capable of housing colonies of insects for multiple generations, these properties may offer a mechanical advantage over the basal hymenopteran silk [6]. However, these properties do not offer a selective advantage to the individual and hence cannot explain the retention of the genes for this new building material in the non-social ancestors of the Vespoidea, Apoidea and Formicoidea.

In an effort to understand the selective advantage of the silk to the early solitary aculeates we looked more broadly at possible biochemical properties of the material and found that features of the aculeate coiled-coil silk are similar to those of α-helical antimicrobial peptides (α-AMPs) [7,13]. α-AMPs are peptides with broad spectrum microbial killing activity that have been found in most species, from bacteria to mammals. They are cationic (have a positive net charge), making them selective for negatively charged bacterial membranes [13], and are diverse in size and sequence. α-AMPs are generally unstructured in solution and then adopt an amphipathic helical structure within the microbial membranes, creating pores leading to cell lysis and death [14]. Similarly, many peptides from within the aculeate silk proteins have a net positive charge, are unstructured in solution and adopt a coiled-coil structure comprising multiple amphipathic helices at high protein concentrations [15,16].

Previous studies that have investigated the antimicrobial properties of the coiled-coil silk have focused on assessing antifungal activity. These studies found that co-location of silk and ants from *Polyrhachis* [17] or *Oecophylla* [18] genera inoculated with the fungus *Metarhizium* did not lead to higher survival rates of the ants compared to no-silk controls. Here, we use a model bacterial system to investigate if peptides from extant and ancestral aculeate silk protein sequences contain antibacterial properties. We hypothesised that antimicrobial peptides will be found in the silk protein and that this result would support a role for the evolution of this unique silk in the evolution of sociality in these lineages.

## Materials and methods

### Generation of peptides from extant honeybee silk proteins for antimicrobial testing

**Fig 1:**
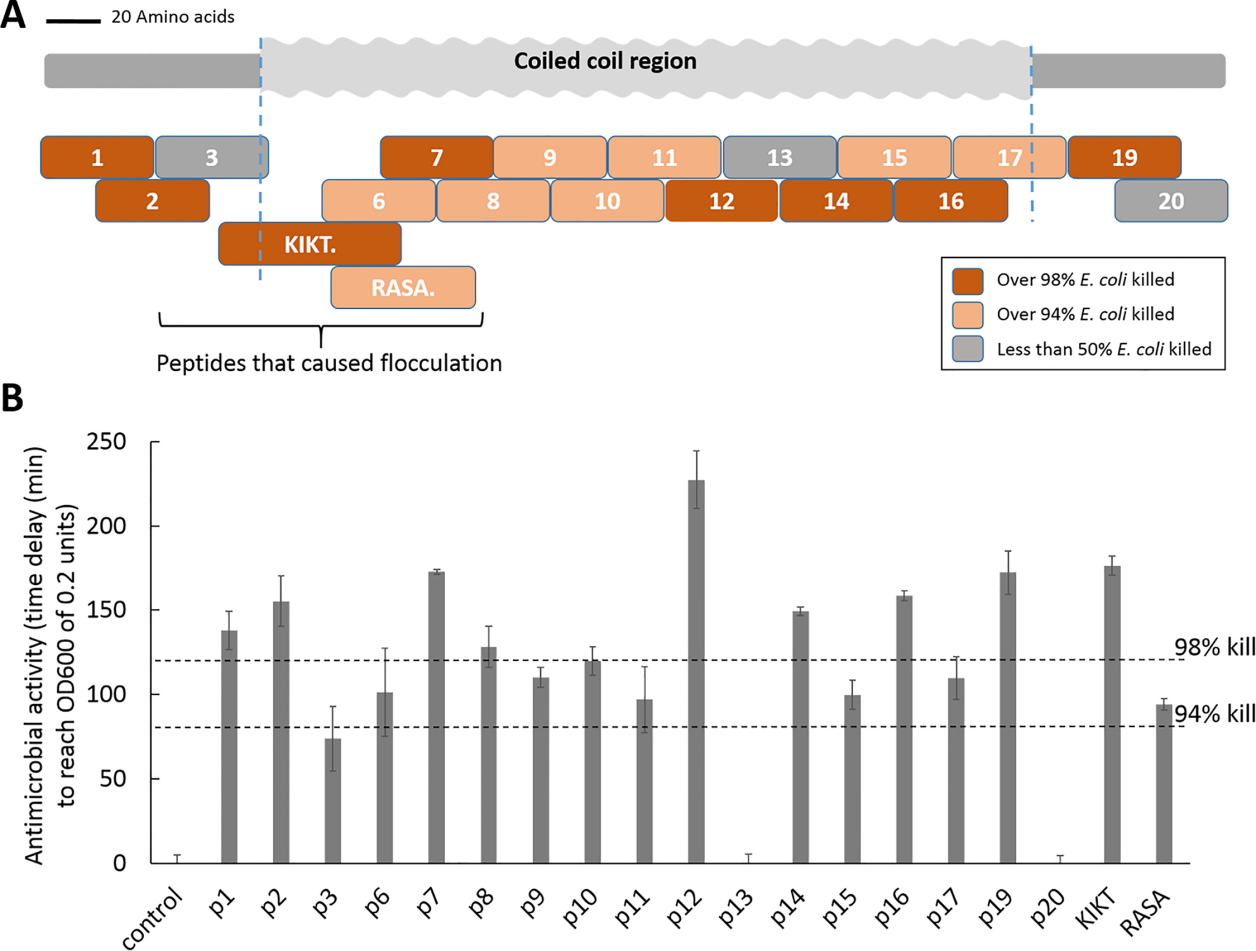
Testing the antimicrobial activity of peptides from the honeybee (*Apis mellifera*) silk protein AmelF3. **A.** Schematic showing where the peptides are located within the silk protein. Colors indicate strength of antimicrobial activity and a bracket identifies which peptides led to flocculation of the bacteria. The predicted coiled-coil region of the protein is indicated. **B.** Comparative antimicrobial activity of the various peptides expressed as the time delay (min) for an *E*. *coli* culture to reach an optical density at 600 nm (OD600) of 0.2 units. Dotted lines show the delay associated with 94 and 98% kill of the population. Error bars show the standard error of the mean calculated from four separate growth cultures.

The relative location of the peptides from the European honeybee (*Apis mellifera*) silk protein AmelF3 (Accession number ACI49702) that were tested for antimicrobial activity are shown in the schematic in Fig 1. We tested 17 consecutive, overlapping peptides (S1 Table) that spanned the entire sequence with the exception of three regions where the peptides could not be generated commercially. These peptides were generated at 75% purity by GL Biochem Ltd (Shanghai, China).

In addition to the overlapping peptides above, we analysed the protein sequence in an attempt to identify the ‘most probable’ antimicrobial peptides based on the following five criteria characteristic of α-helical antimicrobial peptides [13]: a significant proportion of the peptide was within the predicted coiled-coil region (predicted by MARCOIL [19]) and hence had a high propensity to form amphipathic α-helices; the peptide had an overall positive charge of at least +2; the peptide was longer than 22 amino acids, the length required for an α-helical peptide to span a microbial membrane; the peptide was less than 40 amino acids, hence could be made by peptide synthesis; and, the peptide contained at least 50% hydrophobic amino acids, to allow penetration into the microbial membrane. This process identified two overlapping peptides: *RAS ALS AAA SAK AAA ALK NAQ QAQ LNA QEK SLA ALK AQS* (RASA); *KIK TSA SVN AKA AAV VKA SAL ALA EAY LRA SAL SAA ASA KAA AAL KNA* (KIKT). These peptides were generated at 85% purity by Mimotopes (Melbourne, Australia).

### Antimicrobial assays

We compared antimicrobial activity of silk peptides using a laboratory assay based on that of Lok et al. [20] and described by Trueman et al. [21] that uses laboratory strains of the gram negative species, *Escherichia coli* (ATCC 27325). This method is convenient for detecting a wider range of antimicrobial activity than standard minimal inhibitory concentration (MIC) assays [21]. Antimicrobial peptides generally have broad-spectrum activity against a range of microbes. This assay is intended to determine if antimicrobial activity is present and is not intended to mimic the microbial type or load that may be experienced in natural settings.

Briefly, a fresh culture of *E*. *coli* (ATCC 27325) cells were grown in Luria broth to an optical density (600 nm) between 0.1 and 0.2. The cells were pelleted by gentle centrifugation and resuspended in 20 mM Tris, 50 mM NaCl, pH 6.8 at 10^6^ cells.mL^-1^. The cells were then added to the various peptides to reach a final concentration of the peptide of 100 μg.mL^-1^, then the peptide/cell mixture was incubated at 4°C with shaking at 75 rpm overnight. After this treatment, an equivalent volume of double strength Luria broth was added, the culture was incubated at 37°C with shaking at 600 rpm and the optical density of the culture at 600 nm measured on a regular basis. Antimicrobial activity was determined by the delay in time compared to controls for the culture to reach an optical density at 600 nm of 0.2. Control cultures were prepared in the same manner without the presence of peptide. The growing culture was visually inspected every hour to determine flocculation (bacteria clumping together).

The minimal inhibitory concentration (MIC) of peptides with antimicrobial activity was determined by generating a serial dilution of the peptide in water and incubating the dilutions with 10^6^ *E*. *coli* cells in 20 mM Tris, 50 mM NaCl, pH 6.8 with shaking at 75 rpm for 4 h at room temperature. After this treatment, an equivalent volume of double strength Luria broth was added and the cells were incubated for 16 h at 37°C with shaking at 600 rpm. At the end of the incubation the optical density at 600 nm was measured and the lowest concentration to prevent visible growth was recorded as the MIC.

The peptide, KIKT, was sent to the Community for Open Antimicrobial Drug Discovery at The University of Queensland (Australia) where it was tested at 32 μg/mL for antimicrobial activity against pathogenic species of *Escherichia coli* (ATCC 25922), *Klebsiella pneumoniae* (ATCC 700603), *Acinetobacter baumannii* (ATCC 19606), *Pseudomonas aeruginosa* (ATCC 27853), *Staphylococcus aureus* (ATCC 43300), *Candida albicans* (ATCC 90028), and *Cryptococcus neoformans* (ATCC 208821) according to their standard protocols.

For antimicrobial assay all bacteria were cultured in cation-adjusted Mueller Hinton broth at 37°C overnight and then a sample of each culture was diluted 40-fold in fresh broth and incubated at 37°C for 1.5–3 h to give mid-log phase cultures. The optical density (600 nm) of these cultures were determined and the cultures diluted to give a cell density of 5x10^5^ CFU/mL and then added to samples of the peptide to give a final peptide concentration of 32 μg/mL in wells of a 384 well, non-binding surface plates (Fisher Scientific). The peptide/bacterial mixtures were then incubated at 37°C for 18 h without shaking. The effect of the peptide on bacterial growth was determined by measuring absorbance on the culture at 600 nm after the 18 h incubation. The percentage of growth inhibition was calculated for each well, using absorbance from media only as a negative control and growth of the bacteria without the peptide as a positive control on the same plate as references.

Fungi strains were cultured for 72 hrs on Yeast Extract-Peptone Dextrose agar at 30°C. Five colonies were used to generate a yeast suspension. The optical density (530 nm) of the suspension was determined and the culture diluted to the equivalent of 2.5x10^3^ CFU/mL. The yeast suspension was added to the peptides to give a final peptide concentration of 32 μg/mL in wells of a 384 well, non-binding surface plates (Fisher Scientific). Plates were incubated at 35°C for 24 h without shaking. After the incubation period, growth of *C*. *albicans* was determined from increases in absorbance (530 nm) of the culture and *C*. *neoformans* growth was determined by measuring the difference in absorbance between 600 and 570 nm (OD600-570), after the addition of resazurin (0.001% final concentration) and incubation at 35°C for additional 2 h. The percentage of growth inhibition was calculated for each well, using absorbance from media only as a negative control and growth of the fungi without the peptide as a positive control on the same plate as references.

### Ancestral state reconstructions

Ancestral state reconstruction was performed using the Jupyter notebooks that can be found at https://github.com/BenKaehler/gapped. The exact configurations for the following tools can be found there. The nucleotide sequences were translated into protein sequences using PyCogent [22], aligned using MAFFT [23], and translated back into codon alignments using PyCogent. The CNFGTR model of codon evolution ([24] was fitted to the alignment using the maximum likelihood methods in PyCogent. The CNFGTR model was modified to allow gap codons to be treated as a character state with the introduction of two more parameters: the stationary gap probability and the indel transition rate. The modified CNFGTR model is implemented in the new Python package *gapped*, which can be installed from https://github.com/BenKaehler/gapped. Finally, joint ancestral state reconstruction was performed using the algorithm given in Pupko et al. [25]. This algorithm is also implemented in the gapped package and makes joint ancestral state reconstruction possible for any PyCogent model.

**Fig 2:**
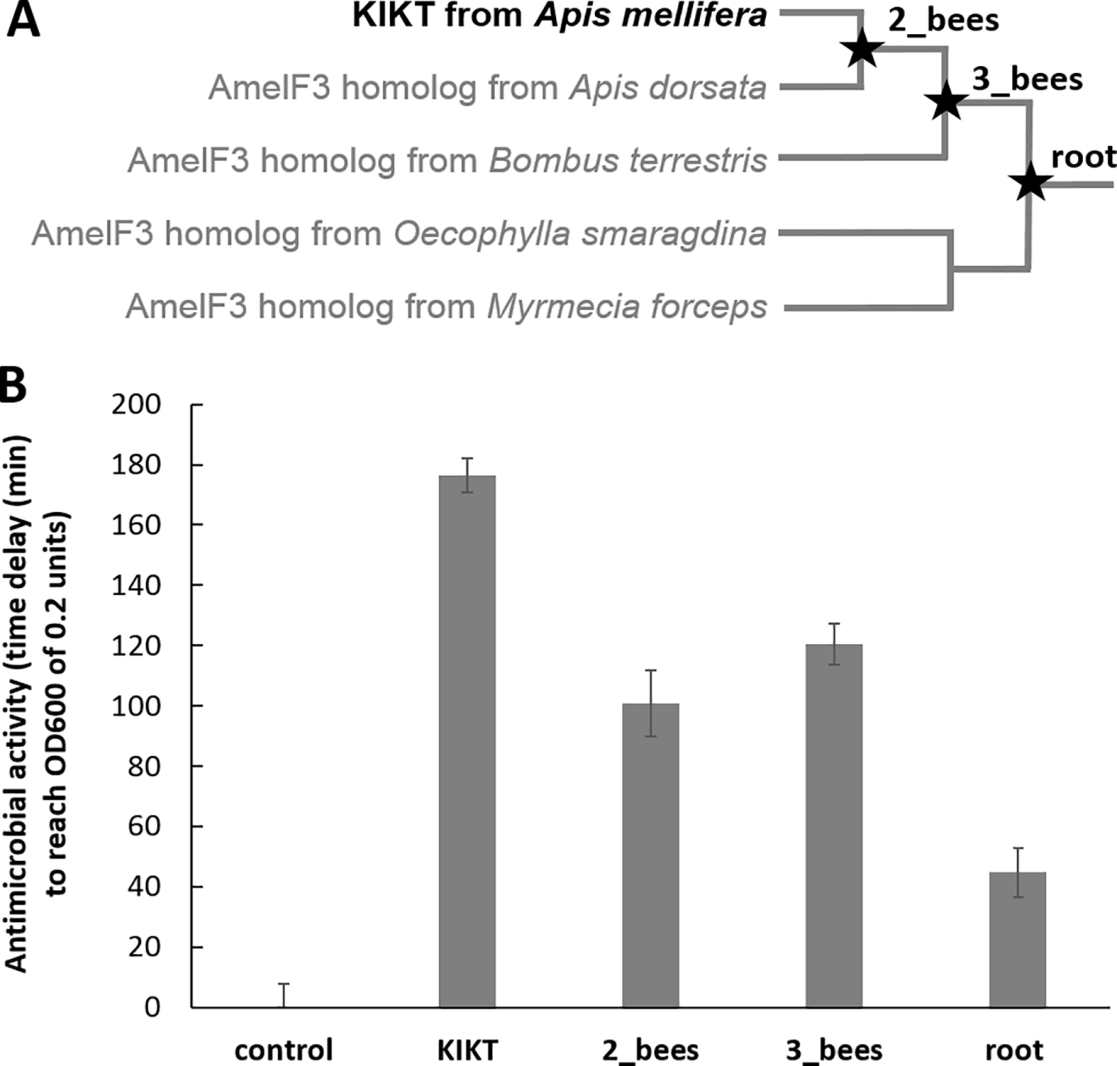
Testing the antimicrobial activity of the KIKT peptide from ancestral and extant sequences homologous to AmelF3 from honeybees. **A.** Phylogenetic tree of bee species within the Hymenopteran suborder Aculeata with black typeface indicating extant and ancestral sequences used in the analysis. Full protein sequences can be found in S2 Table. **B.** Comparative antimicrobial activity of the various peptides expressed as the time delay (min) for an *E*. *coli* culture to reach an optical density at 600 nm (OD600) of 0.2 units. NB. The peptides generated from ancestral sequences contained a higher proportion of hydrophobic residues and precipitated from solution during the assay period. Error bars show the standard error of the mean calculated from four separate growth cultures.

From the ancestral sequences (S2 Table), the KIKT peptide region (see above), which had features of known antimicrobial peptides and demonstrated antimicrobial activity, was selected for experimental analysis of antimicrobial activity. In addition to the KIKT peptide from extant AmelF3, we experimentally tested the antimicrobial activity of the peptides from the ancestral node of the *Apis mellifera* and *Apis dorsata* AmelF3 homologue (KIK ASA GAD AKA SAV VKA SAL ALA EAY LRA SAL SAA ASA KAA AAL K; 2_bees); the ancestral node of 2_bees and the *Bombus terrestris* AmelF3 homlogue (KTK ATA AAD AKA SAM VKA SAL ALA EAY LRA SAA SAA ASA KAA AAV K; 3_bees); and the ancestral node of the 3_bees peptide and the homologous proteins from the ant species *Oecophylla smaragdina* and *Myrmecia forceps* (KAK AIA AAD AKA SAM VKT VAV ALA KAY VRA AAA SAA ASA KAV ATV K; root) (Fig 2A). The F1, F2, and F4 paralogues of from the same five species were used as an outgroup for the purpose of constructing the root sequence.

## Results

### Antimicrobial activity of peptides from silk protein of extant honeybees

The peptides derived from the European honeybee silk protein AmelF3 (Fig 1A) were tested for their ability to prevent growth of *E*. *coli* strain K-12 cells (ATCC 27325) in a laboratory assay. Although this strain of *E*.*coli* is not an environmental pathogen of honeybee, antimicrobial peptides have broad spectrum killing activity and this assay is a convenient method to test for antimicrobial activity.

Of the 19 peptides tested from AmelF3, all except two were able to kill 50% or more of the *E*. *coli* cells in our tests (Fig 1B). Nine of the peptides killed more than 98% of the bacteria. Whilst this does not conform to the standard biomedical definition of ‘antimicrobial’ (ability to prevent all detectable growth of 10^6^ bacterial cells for at least 16 h, which would require killing >99.9999% of the *E*. *coli*), the activity is significant. Further analysis of the growth curves obtained after incubation of *E*. *coli* cells with different concentrations of the KIKT peptide demonstrated an initial increase in optical density (600 nm) that correlated with the amount of peptide present (S1 Fig). This is consistent with earlier studies that show swelling of *E*. *coli* in the presence of antimicrobial peptides [26], rather than indicating microbial growth. Four of the peptides (p3, p6, p7 and RASA) led to flocculation of the bacteria (Fig 1A).

The peptide, KIKT, was sent to the Community for Open Antimicrobial Drug Discovery at The University of Queensland (Australia) where it was tested for antimicrobial activity against a range of pathogenic species using their standard concentrations (32 μg/mL) which are significantly lower than the concentrations found to have activity in against the laboratory *E*. *coli* strains (100 ug/mL) and require the antimicrobial to prevent all microbial growth for at least 16 hours. In our assay, the peptides delayed growth by up to 6 hours. The Community for Open Antimicrobial Drug Discovery tested the peptide against pathogenic strains of *Staphylococcus aureas*, *Escherichia coli*, *Klebsiella pneumoniae*, *Acinetobacter baumannii*, *Pseudomonas aeruginosa*, *Candida albicans* and *Crypotococcus neoformans* using the facility’s standard testing protocols. Growth of all species was similar to controls without peptide, indicating that the peptide did not inhibit growth of these pathogenic species at the tested concentration. Given the conditions, which are designed to identify antimicrobials suitable for further drug development, it is not surprising that no antimicrobial activity was detected.

### Antimicrobial activity of peptides from silk protein of ancestral sequences

We compared the KIKT peptides from the ancestral sequences to that from the extant AmelF3 sequence. All the KIKT homologues had some level of antimicrobial activity compared to controls without peptides (Fig 2). There was a general trend in the level of activity observed, with the extant sequence showing the greatest activity and the most ancestral sequences showing the least activity. Possibly this trend reflects selection for antimicrobial activity within the sequences. On the other hand, the peptides from the ancestral sequences were found to have a low solubility in the assay medium and were observed to precipitate over the course of the experiment. Therefore, the findings may not be a true reflection of the peptides’ activity, due to a decrease in the active peptide concentration over the assay period. The peptides’ insolubility is likely due to the increased level of hydrophobicity in the ancestral peptides: AmelF3 KIKT had 62.5% hydrophobic residues in comparison to 65.2% hydrophobic residues in RootF3 2 bees, 63% hydrophobicity in RootF3 3 bees, and 67.4% hydrophobic residues in the peptide from the ancestral root to AmelF3 sequence. The use of DSMO to solubilise the peptides prior to assay did not improve solubility of the peptides in the incubation solution and significant precipitation was noted during this stage.

## Discussion

### Peptides from extant aculeate silk proteins have antimicrobial activity

In this study, we demonstrate that the honeybee silk protein, AmelF3, harbors antimicrobial peptides. Despite anecdotal descriptions, there is no evidence for antimicrobial activity in the silks of spiders and silkworm [27,28]. The finding of antimicrobial peptides in the coiled-coil silk supports our conjecture that the silk of the Vespoidea, Apoidea and Formicoidea evolved both as a structural material and as an antifouling.

An advantage of having an antifouling mechanism hidden within the silk material is that such a mechanism is likely to target pathogenic species over beneficial species. In order to establish infection, pathogenic species typically interact with their host by releasing proteases, enzymes that hydrolyse protein bonds [29]. Nearly all animals are host to a beneficial microbial population, with honeybees hosting beneficial microbes both within their bodies and within their hives [30,31], at a microbial loading estimated to be 10^4^−10^5^ bacteria/gram of hive [32]. It is speculated that these communities play a role in general hygiene, pathogen inhibition and/or bee bread fermentation/preservation [31,33]. A documented example of this is a *Streptomyces* species found in the brood comb, crop and bee bread. This commensal species produces candicidin [34], a compound that is active against a common honeybee yeast pathogen [31]. In order to preserve these positive microbial interactions, social insects need a mechanism that will target the pathogens but not affect the beneficial species. Integrating the antimicrobial peptides within the silk material is potentially such a mechanism–the peptides will only be released upon proteolytic cleavage of the silk in response to pathogenic species.

Whilst we are not aware of other examples of silks that harbor antimicrobial peptides, there are a number of soluble proteins that contain peptides with antimicrobial activity. Examples include lactoferrin, a glycoprotein widely found in milk, saliva, tears and nasal secretions, which has bactericidal activity both as an intact protein and after pepsin cleavage to release peptides known as lactoferricins [35]. Pepsin hydrolysis of the milk protein casein to short (5–12 amino acid) peptides inhibit growth of *E*. *coli* by up to six logs [36,37]. Lysozyme, an antimicrobial protein widely found in biological fluids and tissues, is cleaved by pepsin under biologically relevant conditions, to generate five antimicrobial peptides [38]. Additionally, hemoglobin, myoglobin and cyctochrome *c* all contain peptides that have antimicrobial activity [39]. Proteolytic degradation of the extracellular matrix material releases antimicrobial peptides [40,41]. It is generally speculated that the antimicrobial peptides within these proteins play a role in maintaining hygiene in the various biological systems where they are found.

### Did evolution of these silks contribute to evolution of sociality?

Sociality has evolved multiple times within the subclade Aculeata, but only within the superfamilies Vespoidea, Apoidea and Formicoidea and not within the sister superfamily Chrysidoidea [2,3]. Coincidently, the genes that encode the coiled-coil silk proteins evolved in the common ancestor of the superfamilies Vespoidea, Apoidea and Formicoidea subsequent to the divergence of the Chrysidoidea [6]. Species from the Chrysidoidea produce a completely different silk, characterised by a β-sheet molecular structure that they use as a structural material to fabricate cocoons. The coincidence of evolution of the coiled-coil silk prior to the evolution of sociality raises the question of whether the silk causally contributed to the evolution of sociality.

It has been suggested that a pivotal evolutionary step towards evolution of eusociality is the use of a domicile that can be provisioned with food to raise immatures, a behaviour associated with many extant non-social aculeates [42]. The ability to maintain hygiene in environments that store food and raise immatures in close proximity is paramount. It is possible that the antimicrobial activity in the silk material contributed to the early aculeates’ ability to maintain sanitation in their domiciles and ultimately to evolution of sociality in this lineage. At later stages of evolution, the various species evolved the plethora of mechanisms used to maintain hygiene in a social context that we know about today.

In this study, we attempted ancestral sequence reconstruction to evaluate this question further. We found evidence that the ancestral sequences did contain antimicrobial activity with the data suggesting an increase in activity over evolutionary time. However, measurements were confounded as the ancestral peptides contained high levels of hydrophobic residues that resulted in their precipitation from solution in our model antimicrobial assay system. The excess of hydrophobic residues in these sequences likely indicates limitations of the current ancestral reconstruction algorithms rather than being a true reflection of the ancestral state of the proteins. Availability of more advanced algorithms will facilitate our ability to conduct this analysis.

### Use of coiled-coil silk as a model for design of new biomaterials

There is a global need for antifouling materials, in particular for antifouling biomaterials. Biomaterials are defined as materials that are introduced into the body to supplement or replace normal body function. Biomaterials include implants such as catheters, prosthetic joints, lenses, stents, renal dialysers, pacemakers and vascular grafts. The use of biomaterials has saved or improved the lives of millions of people and the market for these materials is large and growing—valued at $72.36 billion USD in 2016, with projections of a compound annual growth rate of 16.0% between 2017 and 2021 [43]. However, the major complication risk associated with the use of biomaterials is infection [44] with one in four patients experiencing a device-associated infection [45].

It has long been known that the presence of a biomaterial reduces a patient’s tolerance to infection. In 1957, Elek and Conen [46] found the presence of sutures resulted in a dramatic reduction in the number of *Staphylococcus pyogenes* cells required to produce visible signs of infection. Despite infection being the primary cause of biomaterial implant and device failure, a biomaterial’s resistance to infection has generally been overlooked during past biomaterial development [47]. Healthcare costs associated with biomaterial infections in the USA alone are around $3 billion per annum. As a consequence, there is substantial interest in the medical community to develop new biomaterials that offer better protection against infection.

Infection rates associated with biomaterials are dependent on their type. Biologically derived biomaterials (i.e. collagen or intact extracellular matrix (ECM) materials) are significantly more resistant to infection than synthetic materials [48–52]. A number of mechanisms have been proposed to explain the greater resistance of biologically-derived biomaterials to infection [47]: natural materials promote revascularisation, which stimulates the immune system response [49,53]; as the natural material degrades the level of immune response is reduced [47]; degradation reduces the ability for microbial colonisation [47]; and, degradation of the biologically derived material releases antimicrobial peptides [40,41].

Biomaterials composed of decellularised ECM have been the basis of a large number of commercial products for over a decade [see lists in 54,55]. Many trials have shown superior performance of these materials, compared to their synthetic equivalents. However, their biological origin raises concerns as to their structure and composition, which vary according to the method of production and type and age of the animal from which they were harvested [reviewed in 55]. Furthermore, the structure and composition cannot be tailored for specific needs. In contrast, the coiled-coil silk proteins of aculeates are encoded by comparatively small and non-repetitive genes, making them ideal for recombinant production in artificial systems [56].The fact that this is a naturally occurring material that can be tailored for specific needs [57], coupled with the natural antimicrobial properties incorporated into its structure suggests further examination of the molecular properties of this silk has considerable potential for biomaterial engineering.

## Supporting information

S1 Fig **A**. Growth curves obtained after incubation of *E*. *coli* cells with the KIKT peptide from the honeybee silk protein AmelF3 at concentrations from 12.5–200 μg/mL. **B**. Increases in optical density seen within the first hour of incubation of *E*. *coli* cells with the KIKT peptide from the honeybee silk protein AmelF3. Error is standard error of the mean.
(DOCX)

S1 Table Sequence and properties of the peptides from extant *Apis mellifera* silk protein sequence used in this study. (DOCX)

S2 Table Sequence of ancestral sequences predicted in this study and the extant sequences used in their construction. (DOCX)
