# Supplementary material for: Did aculeate silk evolve as an antifouling material?
Source: PLoS One. 2018 Sep 21;13(9):e0203948. doi: 10.1371/journal.pone.0203948 (PMC6150510; doi:10.1371/journal.pone.0203948)
Supplement: S1 Fig — A. Growth curves obtained after incubation of E. coli cells with the KIKT peptide from the honeybee silk protein AmelF3 at concentrations from 12.5–200 μg/mL. B. Increases in optical density seen within the first hour of incubation of E. coli cells with the KIKT peptide from the honeybee silk protein AmelF3. Error is standard error of the mean. (DOCX) [file pone.0203948.s001.docx]

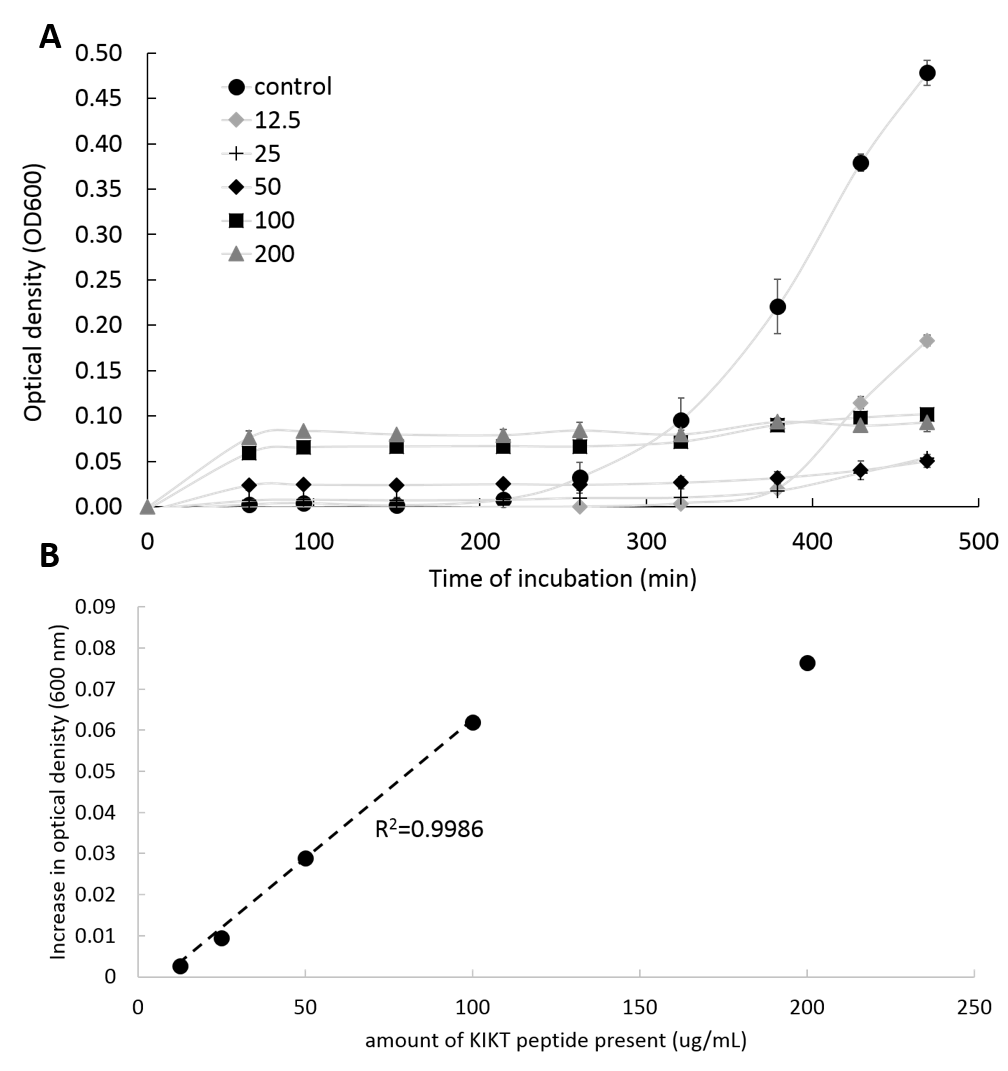


**Supplementary Figure 1.** **A**. Growth curves obtained after incubation of *E. coli* cells with the KIKT peptide from the honeybee silk protein AmelF3 at concentrations from 12.5 – 200 µg/mL. **B**. Increases in optical density seen within the first hour of incubation of *E. coli* cells with the KIKT peptide from the honeybee silk protein AmelF3. Error is standard error of the mean.
