# Supplementary material for: Did aculeate silk evolve as an antifouling material?
Source: PLoS One. 2018 Sep 21;13(9):e0203948. doi: 10.1371/journal.pone.0203948 (PMC6150510; doi:10.1371/journal.pone.0203948)
Supplement: S1 Table — (DOCX) [file pone.0203948.s002.docx]

**Supplementary Table 1.** Sequence and properties of the peptides from extant *Apis mellifera* silk protein sequence used in this study.

| **Peptide** | **Peptide sequence** | **Predicted % helical structure*** | **Estimated charge at pH 7.0*** | **% hydrophobic residues** |
| --- | --- | --- | --- | --- |
| 1 | VEEFKSSATEEVISKNLEVDLLKNVDTSAK | 30 | 37 | 27 |
| 2 | KNLEVDLLKNVDTSAKRRENGAPVLGKNTL | 36 | 37 | 30 |
| 3 | RRENGAPVLGKNTLQSLEKIKTSASVNAKA | 40 | 37 | 37 |
| 6 | YLRASALSAAASAKAAAALKNAQQAQLNAQ | 80 | 57 | 33 |
| 7 | AALKNAQQAQLNAQEKSLAALKAQSEEEAA | 70 | 47 | 30 |
| 8 | EKSLAALKAQSEEEAASARANAATAATQSA | 73 | 47 | 30 |
| 9 | SARANAATAATQSALERAQASSRLATVAQN | 73 | 47 | 40 |
| 10 | LERAQASSRLATVAQNVASDLQKRTSTKAA | 73 | 40 | 37 |
| 11 | VASDLQKRTSTKAAAEAAATLRQLQDAERT | 83 | 40 | 30 |
| 12 | AEAAATLRQLQDAERTKWSANAALEVSAAA | 43 | 53 | 23 |
| 13 | KWSANAALEVSAAAAAAETKTTASSEAANA | 63 | 53 | 30 |
| 14 | AAAETKTTASSEAANAAAKKAAAIASDADG | 50 | 50 | 27 |
| 15 | AAKKAAAIASDADGAERSASTEAQSAAKIE | 50 | 47 | 23 |
| 16 | AERSASTEAQSAAKIESVAAAEGSANSASE | 50 | 40 | 38 |
| 17 | SVAAAEGSANSASEDSRAAQLEASTAARAN | 53 | 43 | 37 |
| 19 | VAAAVGDGAIIGLGEEAGAAAQLLAQAKAL | 46 | 63 | 23 |
| 20 | EAGAAAQLLAQAKALAEVSSKSENIEDKKF | 53 | 47 | 23 |

*Predicted by YASPIN prediction at <http://www.ibi.vu.nl/programs/yaspinwww/>; charge at pH 7.0 from protein calculator v3.4 (http://protcalc.sourceforge.net/cgi-bin/protcalc).
