# Supplementary material for: Did aculeate silk evolve as an antifouling material?
Source: PLoS One. 2018 Sep 21;13(9):e0203948. doi: 10.1371/journal.pone.0203948 (PMC6150510; doi:10.1371/journal.pone.0203948)
Supplement: S2 Table — (DOCX) [file pone.0203948.s003.docx]

**Supplementary Table 2.** Sequence of ancestral sequences predicted in this study and the extant sequences used in their construction.

| **Protein name used in Figure 2** | **Protein sequence** |
| --- | --- |
| root | MKIPAILVTSLLTWGLASAGVLELSSATASASASSENLLKNVVMSAISKVNGAPKLGMGM  KASALAKAKAIAAADAKASAMVKTVAVALAKAYVRAAAASAAASAKAVATVKEAEQAQLI  AEEKAIAASKALSEAVEASVRADAAAAATMAAIERAQASARAATAAQAKASDQAKSANSK  AAAEAAAALRAEEDAAETKWSAAAAVAAAAAAAAVEAKATASSEATGDAAGEARAAAAEA  NAAQASAAAEAQSAAQIEDKAGADRSAASASADSRAALAEAAAAAKAAAAAAVRDGAIIG  LGEDASSAAQALAQVKALASADASAESGTKEWVW |
| 3_bees | MQIPAILVTCLLTWGLVHAGVVEFSSATESVLVEKLLLKNVETSAKRKENGAPKLGKSTA  AALAKTKATAAADAKASAMVKASALALAEAYLRASAASAAASAKAAAAVKEAKQAQLIAQ  EKALAALKAQSEEEAASARADAAAAATVSALERAQASSRAATAAQDIASDLEKRASTKAA  AEAAATLRAEQDAAQTKWSAASAVEAAAAAAAVEAKATASSEATGAAASKAAALAADANG  AEASAAAEAQSAAQIEGIAAAEGSANSASEDSRAAQLEASAAAKATAAAAVGDGAIIGLG  QDASAAAQALAQVKALAEASAKSGTIEKDKW |
| 2_bees | MQIPTFLVICLLTSGLVHAGVEEFKSSATEEVIGKKLEVDLLKNVDTSAKRKENGAPVLG  KNTFKSLEKIKASAGADAKASAVVKASALALAEAYLRASALSAAASAKAAAALKNAQQAQ  LIAQEKALAALKAQSEEEAASARANAAAAATQSALERAQASSRIATAAQDVASDLQKRTS  TKAAAEAAATLRQSQDAEQTKWNAKSALEASAAAAAAETKTTASSEAASAAAKKAAAIAS  DADGAERSASTEAQSAAKIESVAAAEGSANSASEDSRAAQLEASAAARANVAAAVGDGAI  IGLGQDAGAAAQLLAQAKALAEVSSKSENIEDKKF |
| AmelF3 from *Apis mellifera* | MQIPTFVAICLLTSGLVHAGVEEFKSSATEEVISKNLEVDLLKNVDTSAKRRENGAPVLG  KNTLQSLEKIKTSASVNAKAAAVVKASALALAEAYLRASALSAAASAKAAAALKNAQQAQ  LNAQEKSLAALKAQSEEEAASARANAATAATQSALERAQASSRLATVAQNVASDLQKRTS  TKAAAEAAATLRQLQDAERTKWSANAALEVSAAAAAAETKTTASSEAANAAAKKAAAIAS  DADGAERSASTEAQSAAKIESVAAAEGSANSASEDSRAAQLEASTAARANVAAAVGDGAI  IGLGEEAGAAAQLLAQAKALAEVSSKSENIEDKKF |
| AmelF3 homolog from *Apis dorsata* | MQIPTLLVICLLGISGLVHAGVEEFKATEEVIGKKLEVDLLKNVDTSAKRKENGAPVLGK  NIFKSLEKIKASAGADAKTSAVVKASALALAEAYLRASALSAAASAKAAAALKNAQQAQL  VAQEKALAALKAQSEEEAASARANAAAAATQSAVERAQASSRIAMAAQDVASDLQKRTST  KAAAEAAATLRQSQDAEQTKWNAKSALEASAAAAAAETKTTASSEAASAAAKKAAAIASD  ADGAERSASTEAQSAAKIESVAAAEGSANSASEDSQAAQLEASAAARANVAAAIGDGAIS  GLGQDAGAAAQLLAQAKALAEVSSKSENIEDKKF |
| AmelF3 homolog from *Bombus terrestris* | MQIPAIFVTCLLTWGLVHAGSVELGAPKQESVLVEQLLLKNVETSAKRKENGAPKLGEST  AAALASTKATAAAEAKASAKVKASALALAEAFLRASAAFAAASAKAAAAVKEATQAQLLA  QEKALIALKTQSEQQAASARADAAAAAAVSALERAQASSRAATTAQDISSDLEKRVATSA  AAEAGATLRAEQSAAQSKWSAALAAQTAAAAAAIEAKATASSESTAAATSKAAVLTADTS  SAEAAAAAEAQSASRIAGTAATEGSANWASENSRTAQLEASASAKATAAAAVGDGAIIGL  ARDASAAAQAAAEVKALAEASASLGASEKDKK |
| AmelF3 homolog from *Oecophylla smargdina* | MKIPAILVTSFLAWGLASGGVPKELGTSISSASASASASASATASSSSKNVHLLPLKSEH  GIVIDKSKFNIRKVVLSAIDEINGAPNIGLGLKQVSLALAKAQASAQSSAEALAIIKKIV  ALLISAYVRAAEAAARASAEALATVRAAEQAQKIAEAKGRAAAEALSELVEASQKADAAA  AGTTDAIERTYQDARAATSAQTKASGEAENANRNAAATLAAVLSIAKAASGQGGTRAAVD  AAAAAAAAAALHAKANAVSQATSKAAAEARVAAEEAASAQASASASAQLTAQLEEKVSAD  QQAASASTDTSAAIAEAEAAALASTVNAINDGVVIGLGNTASSSAQASAQASALARAKNA  RPKIKGWYKIGGATSASASASASASAQSSSQGLVY |
| AmelF3 homolog from *Myrmecia forceps* | MKIPAILVTSFLAWGLASGNLLKESKASASASASASARASGKKNLHVLPLPKKSEHGIVI  DKSVFDIKDVVLSAVDEINGAPKLGLGWKKVSMGVERAEANAAAAAEALAMIKKIAMARS  SAYVQAAWASAQASADALASARVAQASQEAAEAKGRAASEALSRAIEASSRADAAAAATL  DAMDRTMENARAANAAQTQASGQAENANRSAAAILAALLRIAEASALNNEAAVNAAAAAA  AASALQAKANAASQATARAAGQASTAAEEAQSAQEAADKNAELTTVMLEKASADQQAASA  RADYYTASTEAEAAAQASAINALRDGIVVGMGNDAGASAQAMAQVEALARASEHKALGEK  KKGLVWGYGSKGSSSASASASASAEASSRLGKDW |
